# Bone marrow stromal and anterior cruciate ligament remnant cell co‐culture‐derived extracellular vesicles promote cell activity in both cell types

**DOI:** 10.1111/jcmm.70049

**Published:** 2024-09-01

**Authors:** Sung‐Yen Lin, Shun Cheng Wu, Zi‐Miao Liu, Paul Pei‐Hsi Chou, Chunfeng Zhao, Mei‐Ling Ho, Cheng‐Chang Lu

**Affiliations:** ^1^ Department of Orthopedics Kaohsiung Medical University Gangshan Hospital Kaohsiung Taiwan; ^2^ Department of Orthopedics Kaohsiung Medical University Hospital Kaohsiung Taiwan; ^3^ Department of Orthopedics, School of Post‐Baccalaureate Medicine, College of Medicine Kaohsiung Medical University Kaohsiung Taiwan; ^4^ Regenerative Medicine and Cell Therapy Research Center Kaohsiung Medical University Kaohsiung Taiwan; ^5^ Orthopaedic Research Center Kaohsiung Medical University Kaohsiung Taiwan; ^6^ Department of Nursing Asia University Taichung Taiwan; ^7^ Department of Orthopedics, School of Medicine, College of Medicine Kaohsiung Medical University Kaohsiung Taiwan; ^8^ Biomechanics & Tendon and Soft Tissue Biology Laboratories, Division of Orthopedic Research Mayo Clinic Rochester Minnesota USA; ^9^ Department of Orthopedics, Kaohsiung Municipal Siaogang Hospital Kaohsiung Medical University Kaohsiung Taiwan

**Keywords:** anterior cruciate ligament, bone marrow stromal cells, extracellular vesicle, tenogenesis

## Abstract

The significance of anterior cruciate ligament (ACL) remnants during reconstruction remains unclear. Co‐culturing ACL remnant cells and bone marrow stromal cells (BMSCs) may reduce apoptosis and enhance hamstring tendon activity. This study investigated whether extracellular vesicles (EVs), which facilitate cell–cell interactions, act as the active components, improving graft maturation in this co‐culture. The effects of EVs on cell viability, proliferation, migration and gene expression in the rabbit ACL remnant cells and BMSCs were assessed using control (BMSC‐only culture), co‐culture (ACL remnant cells and BMSCs, CM) and co‐culture without EVs (CM ∆ EVs) media. EVs were isolated from control (BMSC‐EV) and co‐culture (CM‐EV) media and characterized. CM significantly enhanced the proliferation, migration and expression of transforming growth factor (*TGF‐β*)‐, vascular endothelial growth factor (*VEGF*)‐, collagen synthesis‐ and tenogenesis‐related genes. However, CM‐induced effects were reversed by the CM ∆ EVs treatment. CM‐EV treatment exhibited higher potential to enhance proliferation, migration and gene expression in the ACL remnant cells and BMSCs than BMSC‐EV and non‐EV treatments. In conclusion, EVs, secreted under the coexistence of ACL remnant cells and BMSCs, primarily increase the cell viability, proliferation, migration and gene expression of collagen synthesis‐, *TGF‐β*‐, *VEGF*‐ and tenogenesis‐related genes in both cell types.

## 1 INTRODUCTION

The anterior cruciate ligament (ACL) that connects the thighbone (femur) to the shinbone (tibia) plays a crucial role in knee stability during daily activity and exercise. ACL injury frequently occurs during non‐contact sports activities, such as basketball, volleyball and football,^1^, ^2^, ^3^ and can lead to persistent pain and instability. Physical therapy and sport braces are the primary conservative treatment methods to restore mobility and knee function. However, ACL reconstruction may be required depending on the severity of the injury or the presence of other comorbidities, such as an intraarticular ligament or meniscus injury. The standard procedure for ACL reconstruction involves passing a tendon graft through a drilled tibia and femoral bone tunnel. The graft healing process comprises successive stages, including graft necrosis, vascularization, recellularization, remodelling and maturation.^4^, ^5^ Nevertheless, despite advancements in surgical techniques, enhancing graft maturation and preventing graft retear following ACL reconstruction remain significant challenges.

Remnant preservation in ACL reconstruction improves graft maturation.^6^, ^7^ However, whether ACL remnant preservation offers substantial benefits remains debatable within the medical community. For instance, Song et al.^8^ highlighted the poor tissue structure and limited capability to enhance graft maturation with no biomechanical and biological advantages. Conversely, other studies suggest that ACL remnants preserve stem cells, native fibres and proprioceptive nerves, which may significantly affect implanted graft maturation.^9^, ^10^, ^11^ In the intraarticular microenvironment, the ACL remnant is coated with bone marrow cells released from the bone tunnel, and the implanted graft is surrounded by the ACL remnant/bone marrow mixture. Although the intricate relationship between ACL remnants and bone marrow cells in the intraarticular microenvironment plays a crucial role in graft maturation, the exact mechanism remains unknown. Lu et al.^12^ first demonstrated that the ACL remnants upregulate the proliferation and gene expression associated with collagen synthesis and the tenogenesis of bone marrow stromal cells (BMSCs). Further, ACL remnant cell/BMSC co‐culture medium could attenuate the apoptosis and increase the activity of the hamstring tendon and tenocyte.^13^ However, the effect of ACL remnant cell/BMSC co‐culture medium on feedback to the ACL remnant cells and BMSCs is unknown, and the active component of the co‐culture medium involved in graft maturation remains elusive. Extracellular vesicles (EVs), which are secreted by cells to facilitate cell–cell interactions, contain abundant intercellular communication agents, such as mRNAs, microRNAs, lipids and proteins, which regulate the target cell activities.^14^, ^15^ Notably, EVs derived from stem cell culture medium exhibit the regenerative potential to regenerate the injured tissue.^16^, ^17^, ^18^

Therefore, we hypothesized that the ACL remnant cell/BMSC co‐culture medium, simulating the intraarticular microenvironment of ACL reconstruction, positively affect and upregulate ACL remnant cell and BMSC activities. We also investigated whether EVs, as the key active components, modulate the effects of the intraarticular microenvironment on effective graft maturation following ACL reconstruction. To test this hypothesis, in this study, we explored the role of EVs derived from ACL remnant cell/BMSC co‐culture medium in ACL reconstruction on ACL remnant cells and BMSCs bioactivities. The present study reveals the intricate interactions between ACL remnant cells, BMSCs and EVs and provides critical insights into the understanding of graft maturation in ACL reconstruction procedures.

## 2 MATERIALS AND METHODS

### 2.1 Experimental design

In this study, we introduced the direct co‐culture system of ACL remnant cells and BMSCs to simulate the intraarticular microenvironment of ACL reconstruction. The ACL remnant cells and BMSCs were harvested from six skeletally mature New Zealand male rabbits, as described previously.^13^ All animal experiments, tissue harvesting protocols and cell culturing were performed following the procedures described by Lu et al.^13^ The third passage of ACL remnant cells (from the ACL‐cut 4‐week rabbit) and BMSCs was used in this study. All animal protocols were approved by the Institutional Animal Care and Use Committees (Kaohsiung Medical University; approval number KMU‐107193).

**FIGURE 1:**
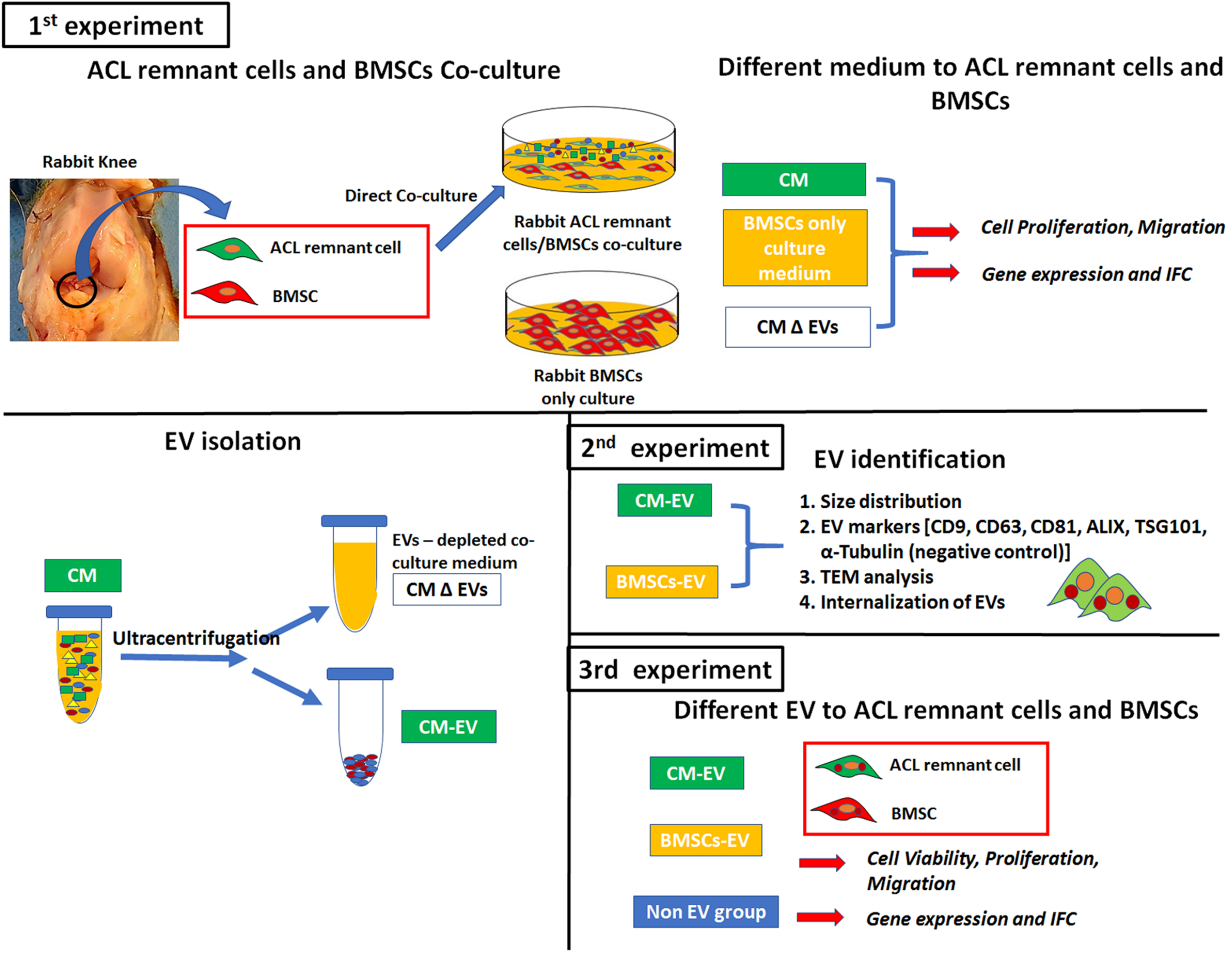
Experimental design of the study. ACL, anterior cruciate ligament; BMSC, bone marrow stromal cell; CM, co‐culture medium; EV, extracellular vesicle.

Figure 1 illustrates the experimental design followed in this study. First, the cells were divided into three groups depending on their medium of growth—control medium (BMSC‐only culture), co‐culture medium (ACL remnant cell and BMSC direct co‐culture, CM) and co‐culture medium without EVs (CM ∆ EVs)—to investigate the effects of ACL remnant cell and BMSC co‐culture on proliferation, migration and expression of collagen I and III (*COL‐I* & *III*), transforming growth factor (*TGF‐β*), vascular endothelial growth factor (*VEGF*) and tenogenic genes (*Scx*, *TNC*). Next, the EVs were isolated from the control (BMSC‐EV) and co‐culture medium (CM‐EV) and characterized based on size, morphology and expression of biological markers. Finally, the effects of EVs on cell activity and gene expression were assessed in ACL remnant cells and BMSCs after BMSC‐EV and CM‐EV treatment compared with those of the non‐EV treatment group.

### 2.2 Preparation of ACL remnant cell/BMSC direct co‐culture and BMSC‐only culture media

The ACL remnant cells (1.5 × 10^5^ cells) and BMSCs (1.5 × 10^5^ cells) were directly co‐cultured (co‐culture medium; CM) in 10 mL medium (low‐glucose DMEM, 10% exosome‐depleted fetal bovine serum [FBS; Cat. No. A2720801, Gibco, Thermo Fisher Scientific, Waltham, MA, USA], and 1% antibiotics [penicillin/streptomycin]) using a 10‐cm culture dish at 37°C and 5% CO_2_. For the control group, only BMSCs (3 × 10^5^ cells) were cultured using the same medium and culture condition. The BMSC‐only culture (control) and co‐culture media were collected every 2 days for 6 days.

### 2.3 Comparison of cell proliferation, migration and expression of genes in BMSC‐only culture medium, CM and CM ∆ EVs

#### 2.3.1 Cell proliferation and migration

The cell proliferation ratio of the ACL remnant cells and BMSCs in response to control medium (*n* = 6), CM (*n* = 6) and CM ∆ EVs (*n* = 6) were measured using a Click‐iT EdU Cell Proliferation Kit (Cat. No. 1906238, Thermo Fisher Scientific, Waltham, MA, USA) following the manufacturer's instructions.^12^, ^13^

To investigate the change in cell migration rate following different media treatments to the ACL remnant cells and BMSCs (*n* = 6, each), transwell and scratch migration tests were employed. The transwell assay was performed using a 6.5‐mm transwell chamber with a pore size of 8 μm (Cat. No. 3422, Costar, Corning Inc., Corning, NY, USA).^12^, ^13^ The migrated cells were fixed by 4% formaldehyde for 15 min, washed with PBS twice and added the methanol 10 min. Then, the migrated cells were stained with 0.1% crystal violet for 30 min. The migrated cells stained in purple were counted under a microscope. The relative migration rate was calculated as the ratio of the migration cells in each treatment group compared to that of the control group (% of the control). The scratch migration test was assessed by observing the closure of the scratch gap at regular intervals (12, 24, 36 and 48 h) under a microscope (Leica DMI6000B; Leica Microsystems, Germany).^12^, ^13^

#### 2.3.2 Expression of COL‐I & III, TGF‐β, VEGF and tenogenic markers (Scx, TNC)

Total RNA from the ACL remnant cells and BMSCs treated with control medium (*n* = 6), CM (*n* = 6) and CM ∆ EVs (*n* = 6) was extracted using RNAzol reagent (Cat. No. RN‐190, Molecular Research Center, Cincinnati, OH, USA). Approximately 2 μg total RNA was reverse‐transcribed using the Maxima First Strand cDNA Synthesis Kit (Cat. No. K1642, Thermo Fisher Scientific Waltham, MA, USA) following the manufacturer's instructions.^13^ The primers, cycling conditions and quantification methods were as described previously.^13^ The primers used for cDNA synthesis are presented in Table S1.

#### 2.3.3 Immunofluorescence staining of COL‐I & III, TGF‐β and VEGF

The ACL remnant cells and BMSCs treated with control medium (*n* = 6), CM (*n* = 6) and CM ∆ EVs (*n* = 6) were assessed for the immunofluorescence expression of COL‐I & III, TGF‐β and VEGF. The primary antibodies, fluorescent secondary antibodies, and the staining procedure were performed as described previously.^13^, ^19^ The primary antibody used in this study are as follows: anti‐COL‐I (1:200, Cat. No. ARG21965, Arigo Biotechnology, Hsinchu City, Taiwan), anti‐COL‐III (1:200, Cat. No. ARG20786, Arigo Biotechnology), anti‐TGF‐β: (1:200, Cat. No. ARG10002, Arigo Biotechnology) and anti‐VEGF (1:200, Cat. No. ARG10513, Arigo Biotechnology). The secondary fluorescence antibodies used in this study were CoraLite594 conjugated donkey anti‐mouse IgG (H + L) (1:250, Cat. No. SA00013‐7, Proteintech, Chicago, IL, USA) and the goat anti‐rabbit IgG (H + L)‐TAMRA (1:250, Cat. No. LDG0047YE, Leadgene Biomedical, Inc. Hsinchu, Taiwan).

### 2.4 Isolation and characterization of EVs from the BMSC‐only culture and co‐culture media

On Day 7 of culture, EVs were isolated from the BMSC‐only culture and CM using a serial centrifugation method.^20^, ^21^ Briefly, the culture medium (500 mL) was centrifuged at 20,000 × *g* for 30 min at 4°C to remove large cell fragments. Subsequently, the supernatant was collected and centrifuged at 120,000 × *g* for 90 min at 4°C to pellet the membrane‐bound vesicles. The medium (supernatant) was removed, and the discarded medium without the membrane‐bound vesicles represented the CM ∆ EVs. The pellet was washed with 10 mL of phosphate‐buffered saline (PBS) and subjected to centrifugation at 120,000 × *g* for 90 min at 4°C. Then, the supernatant was discarded, and the pellet (EVs) was collected and re‐suspended in 1 mL of PBS and filtered with a 0.22‐μm filter membrane.

### 2.5 Characterization of EVs

The BMSC‐EV and CM‐EV were evaluated for size distribution, expression of biological markers, morphology and uptake by the ACL remnant cells and BMSCs.

#### 2.5.1 Size distribution

Particle size analysis was performed using a particle analyser (ZetaView® NTA–Nanoparticle Tracking Video Microscope PMX‐130, Particle Metrix, Ammersee, Germany) following the manufacturer's protocol.

#### 2.5.2 Expression of EV markers (CD9, CD63, CD81, Alix, TSG101 and α‐Tubulin)

Western blotting was performed following the standard protocol. The primary antibodies used in this study, including CD9 (Cat. No. 60232‐1‐1AP), CD63 (Cat. No. 25682‐1‐AP), CD81 (Cat. No. 66866‐1‐AP), Alix (Cat. No. 12422‐1‐AP), TSG101 (Cat. No. 14497‐1‐AP) and the negative control, α‐Tubulin (Cat. No. 66031‐1‐Ig), were all obtained from Proteintech. The labelled proteins were visualized using a ChemiDoc™ XRS imaging system (Bio‐Rad, Hercules, Cal, USA).

#### 2.5.3 Transmission electron microscopy analysis

EV morphology was observed using a transmission electron microscope (Hitachi®, Tokyo, Japan), and the images were captured using a digital camera (Olympus®, Tokyo, Japan).

#### 2.5.4 CM‐EV uptake by the ACL remnant cells and BMSCs

The EV treatment concentration was maintained at 10^10^ particles/10^4^ cells for all experiments. The added EVs were pre‐stained with CM‐DiI (1,1′‐Dioctadecyl‐3,3,3′,3′‐Tetramethylindocarbocyanine Perchlorate; Cat. No. C7000, Thermo Fisher Scientific, Waltham, MA, USA) following the manufacturer's instructions. Briefly, the EVs suspended in 1 mL of PBS were treated with 3 μL of DiI (1 mg/mL) and incubated at 37°C for 5 min, followed by at 4°C for 15 min. Thereafter, EVs were rinsed with PBS, centrifuged at 120,000 × *g* for 90 min at 4°C, re‐suspended in 1 mL serum‐free medium and filtered using a 0.22‐μm filter membrane for further use.

EVs were added to the cells (10^10^ EV particles/10^4^ cells) for 6 h, and the treated cells were washed with PBS and fixed in 4% paraformaldehyde for 10 min. After subsequent washing with PBS twice, 100 μL of 1X phalloidin conjugate working solution (Cat No. ab176753, Abcam, Cambridge, MA, USA) was added to the cells to stain the cytoskeleton. The cell slides were mounted and stained with Hoechst 33342 (Sigma‐Aldrich, St. Louis, MO, USA) and observed using a confocal microscope (Olympus IX‐81‐FV100, Olympus, Tokyo, Japan).

### 2.6 Assessment of the changes in cell viability, migration and gene expression in ACL remnant cells and BMSCs following control (non‐EV), BMSC‐EV and CM‐EV treatments

We investigated the effects of EVs derived from different culture media (BMSC‐EV and CM‐EV) on the viability (MTT assay, Cat. No. M2003, Sigma‐Aldrich, St. Louis, MO, USA), proliferation (Edu assay, *Ki67* gene expression), migration (transwell and scratch migration assays), gene expression (*COL‐I* & *III*, *TGF‐β*, *VEGF*, *Scx*, *TNC*) and immunofluorescence (collagen I and III, TGF‐β, VEGF) of ACL remnant cells and BMSCs. The EV treatment concentration was maintained at 10^10^ particles/10^4^ cells for all experiments. The exosome‐depleted FBS medium‐treated cells were set as a control.

### 2.7 Statistical analysis

All data are presented as the mean ± standard deviation (SD) with three measurements. In this study, the numerical value for the control group was set as 1, and the numerical values of the other experimental groups are presented in reference to the control group. One‐way analysis of variance with a subsequent Tukey post hoc test was used to determine the significant differences in multiple comparisons. All statistical analyses were performed using SPSS software version 20 (IBM, USA). Statistical significance was set at *p* < 0.05.

## 3 RESULTS

### 3.1 Co‐culture medium treatment significantly enhanced the proliferation, migration and gene expression both in ACL remnant cells and BMSCs

**FIGURE 2:**
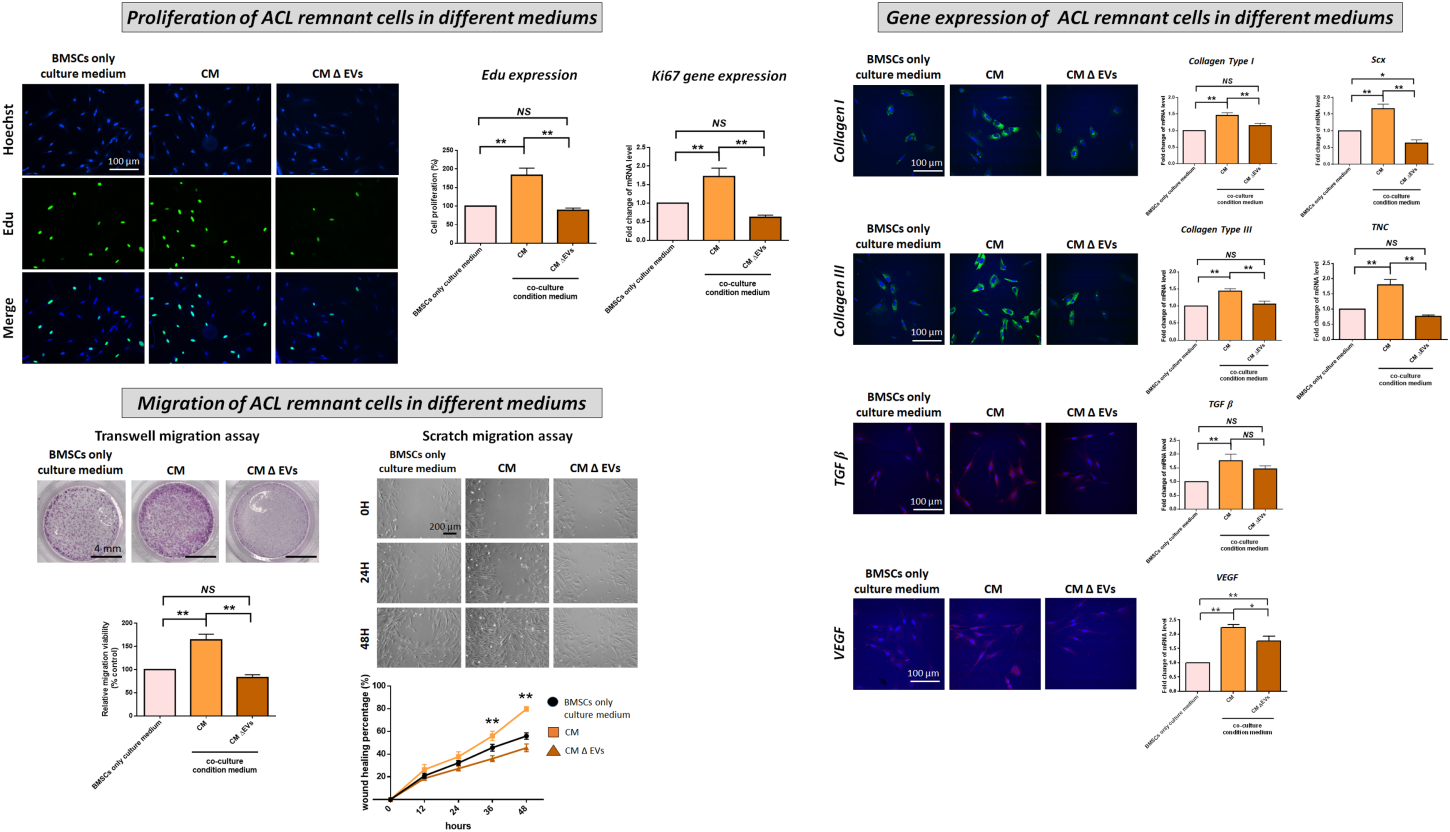
Cell proliferation, migration and expression of collagen I and III‐, TGF‐β‐, VEGF‐ and tenogenic‐related genes in ACL remnant cells following treatment with BMSC‐only culture medium (control), ACL remnant cell/BMSC co‐culture medium (CM) and co‐culture medium without extracellular vesicles (CM ∆ EVs). Migration is evaluated via transwell and scratch migration assays. In the transwell assay, cells that traverse the transwell membrane are stained in purple with crystal violet, and their migration ratio is determined. In the scratch assay, results are presented as the percentage of initial scratch closure. *p < 0.05; **p < 0.01.

**FIGURE 3:**
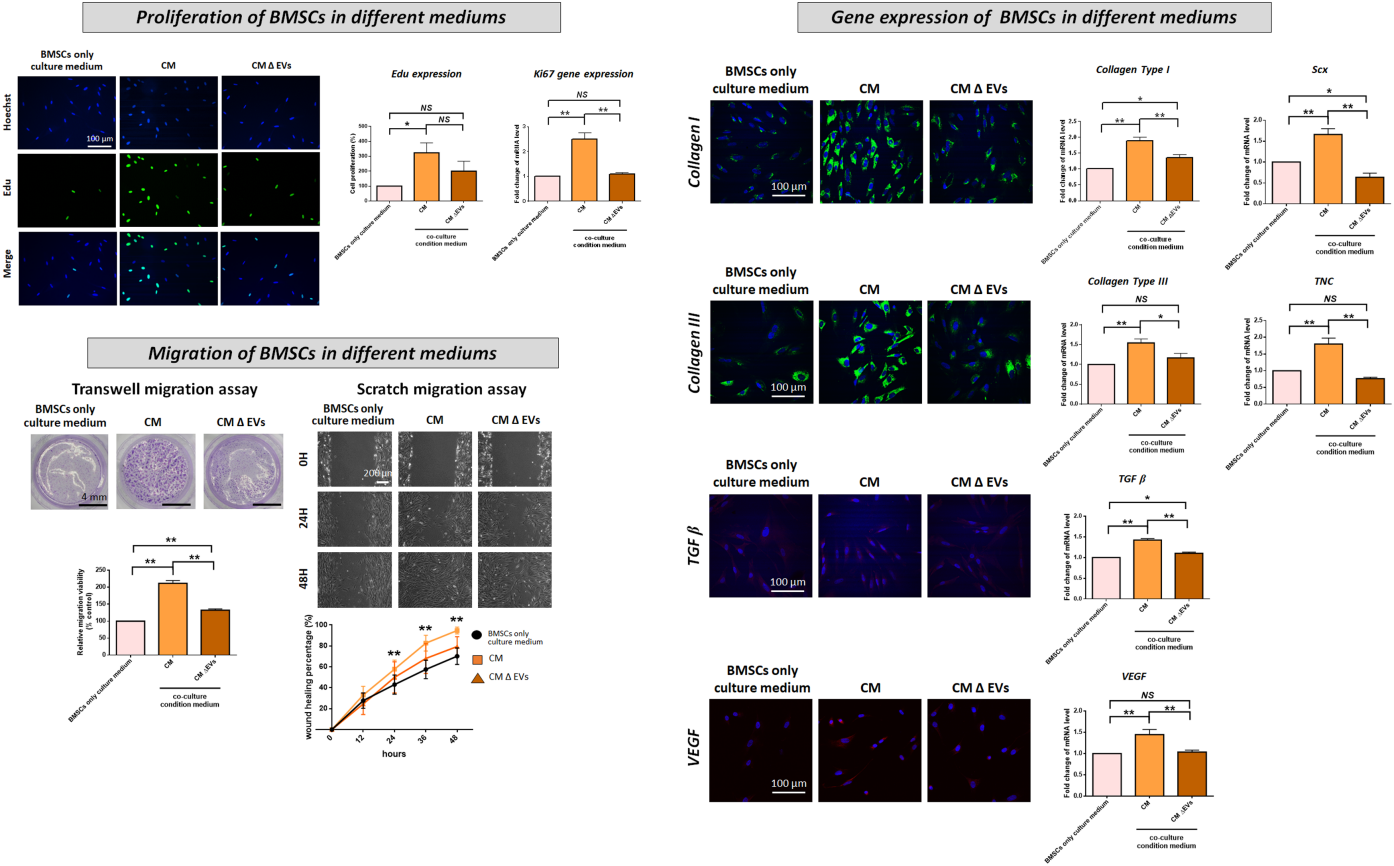
Cell proliferation, migration and expression of collagen I and III‐, TGF‐β‐, VEGF‐ and tenogenic‐related genes in BMSCs following treatment with BMSC‐only culture medium (control), ACL remnant cell/BMSC co‐culture medium (CM) and co‐culture medium without extracellular vesicles (CM ∆ EVs). Migration is evaluated via transwell and scratch migration assays. In the transwell assay, cells that traverse the transwell membrane are stained in purple with crystal violet, and their migration ratio is determined. In the scratch assay, results are presented as the percentage of initial scratch closure. *p < 0.05; **p < 0.01.

The cell proliferation (Edu expression), *Ki67* gene expression and migration capability in both ACL remnant cells (Figure 2) and BMSCs (Figure 3) were significantly increased following CM treatment compared with those after BMSC‐only culture medium treatment. The expression of *COL‐I* & *III*‐, *TGF‐β*‐, *VEGF*‐ and tenogenesis‐related (*Scx*, *TNC*) genes was significantly improved after CM treatment. However, the CM‐induced increases in cell proliferation, migration and gene expression were significantly reversed by the CM ∆ EVs treatment (Figures 2 and 3).

### 3.2 EVs could be isolated from BMSC‐only and co‐culture media, and ACL remnant cells and BMSCs could uptake the EVs

**FIGURE 4:**
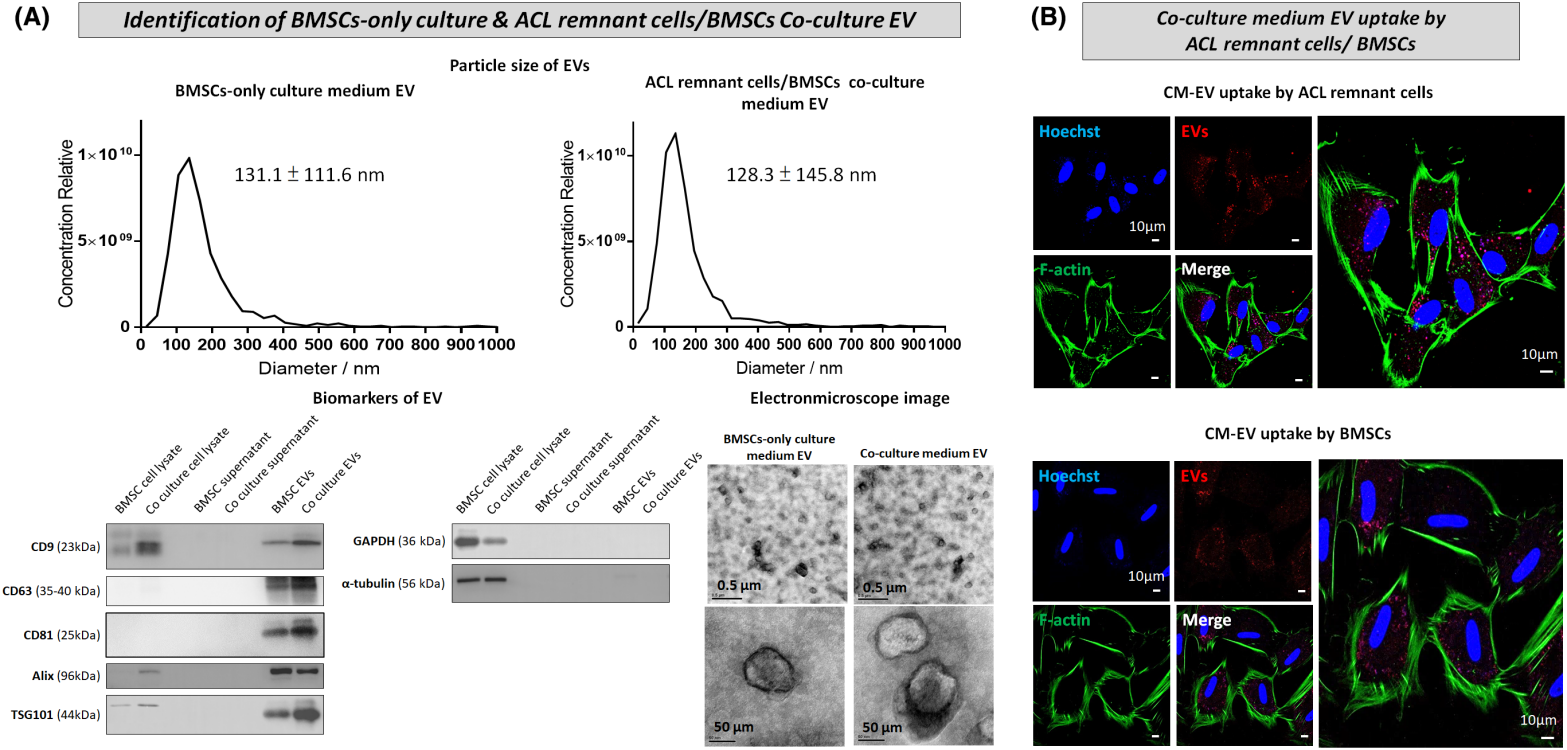
(A) Particle size distribution, expression levels of biomarkers and transmission electron microscopic images of EVs derived from BMSC‐only culture (BMSC‐EV) and ACL remnant cell/BMSC co‐culture medium (CM‐EV). (B) Immunofluorescence pictures show the CM‐EV uptake and internalization by the ACL remnant cells and BMSCs.

EVs from BMSC‐only culture medium and CM were isolated. EVs isolated from BMSC‐only culture medium and CM exhibited an average particle size of 131.1 ± 111.6 and 128.3 ± 145.8 nm, respectively (Figure 4A, upper panel). After purification, the BMSC‐EV and CM‐EV expressed CD9, CD63, CD81, Alix and TSG101, in contrast to the BMSC and Co‐culture media supernatant, which did not show α‐tubulin expression(Figure 4A, lower panel [left]). Moreover, BMSC‐EV and CM‐EV were spherical in shape with central hypodense (light colour) and peripheral hyperdense (dark colour) regions (Figure 4A, lower panel [right]), as determined using microscopic images. Collectively, these results indicated that EVs from both BMSC‐only culture medium and CM were successfully isolated and that isolated EVs met the MISEV 2014 and 2018 criteria.^22^, ^23^, ^24^ Furthermore, the treatment of ACL remnant cells and BMSCs with CM‐EV revealed the potential of these cells to uptake and internalize the EVs (Figure 4B).

### 3.3 Treatment with EVs improved the viability, proliferation, migration and gene expression in ACL remnant cells and BMSCs

**FIGURE 5:**
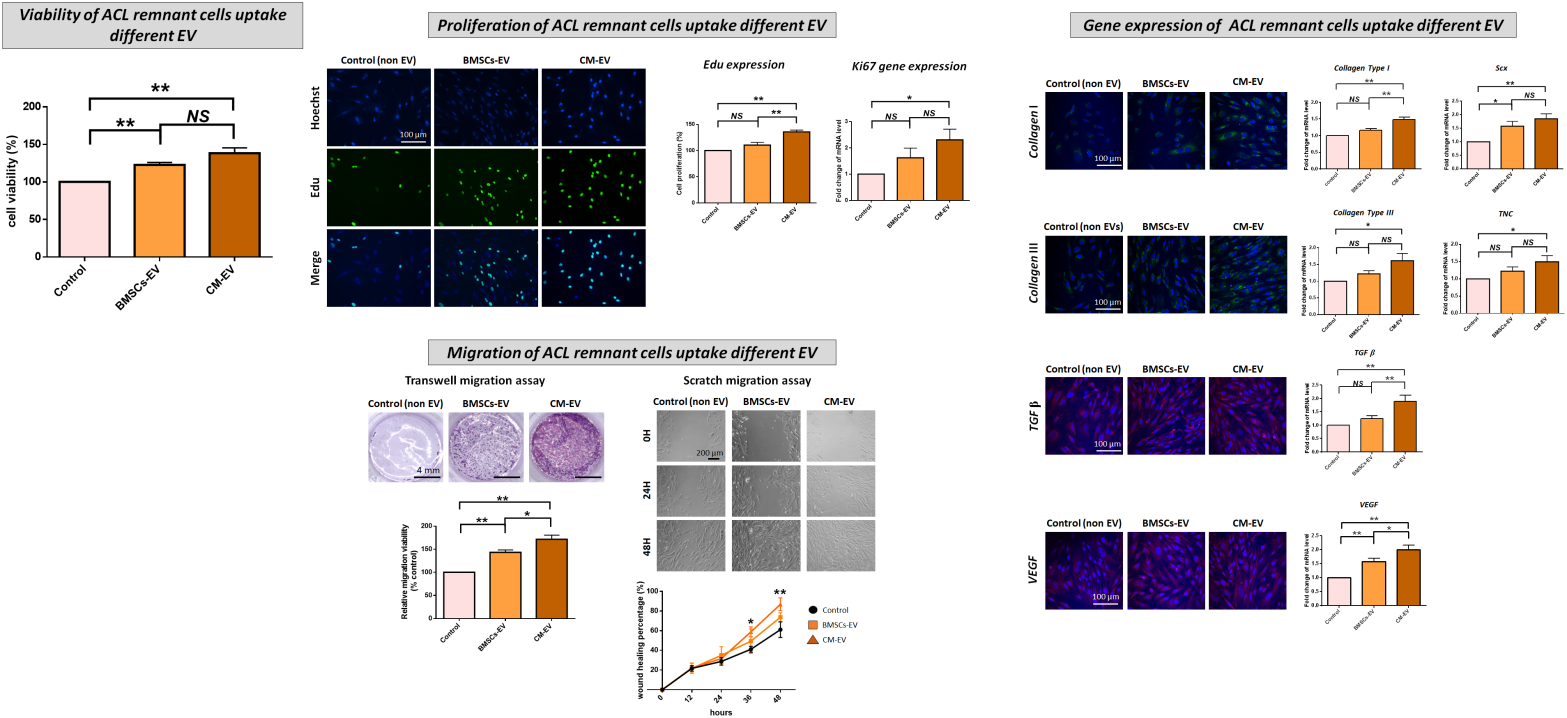
Cell viability, proliferation, migration and expression of collagen I and III‐, TGF‐β‐, VEGF‐ and tenogenic‐related genes in ACL remnant cells following treatment with EVs derived from BMSC‐only culture medium (BMSC‐EV), ACL remnant cell/BMSC co‐culture medium (CM‐EV) and non‐EV medium (control) group. Migration is evaluated via transwell and scratch migration assays. In the transwell assay, cells that traverse the transwell membrane are stained in purple with crystal violet, and their migration ratio is determined. In the scratch assay, results are presented as the percentage of initial scratch closure. *p < 0.05; **p < 0.01.

**FIGURE 6:**
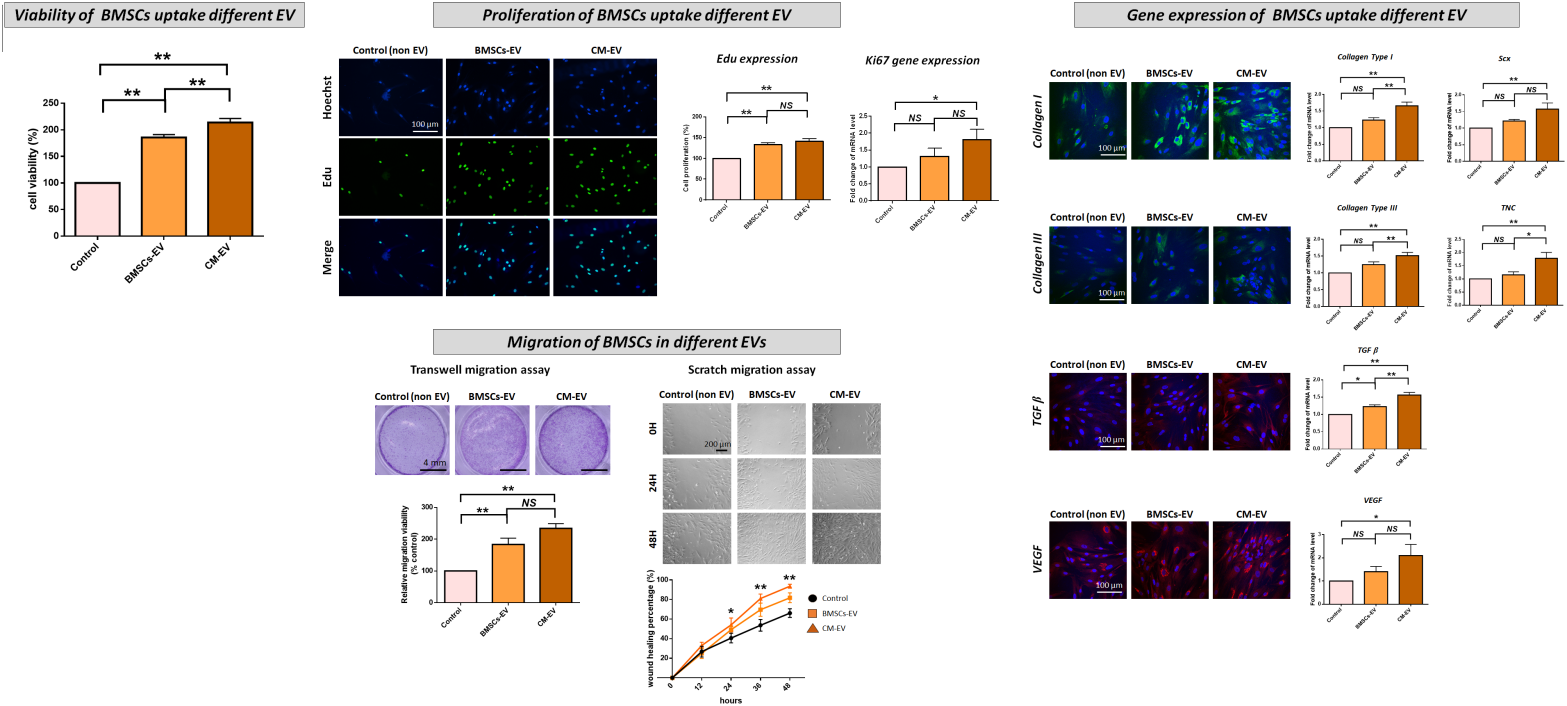
Cell viability, proliferation, migration and expression of collagen I and III‐, TGF‐β‐, VEGF‐ and tenogenic‐related genes in BMSCs following treatment with EV derived from BMSC‐only culture medium (BMSC‐EV), ACL remnant cell/BMSC co‐culture medium (CM‐EV) and non‐EV medium (control) groups. Migration is evaluated via transwell and scratch migration assays. In the transwell assay, cells that traverse the transwell membrane are stained in purple with crystal violet, and their migration ratio is determined. In the scratch assay, results are presented as the percentage of initial scratch closure. *p < 0.05; **p < 0.01.

As shown in Figures 5 and 6, ACL remnant cells and BMSCs treated with BMSC‐EV and CM‐EV showed higher cell viability, proliferation, migration and expression of *COL‐I* & *III*, *TGF‐β*, *VEG*F, *Scx* and *TNC* genes than those treated with non‐EVs (control). These effects were more pronounced in ACL remnant cells treated with CM‐EV than those in the cells treated with BMSC‐EV, with significant differences in proliferation, migration and gene expression of *COL‐I*, *TGF‐β* and *VEGF* (Figure 5). Similarly, all these effects were more pronounced in BMSCs treated with CM‐EV than those treated with BMSC‐EV, with significant differences in cell viability, migration and gene expression of *COL‐I* & *III*, *TGF‐β* and *TNC* (Figure 6).

## 4 DISCUSSION

In this study, we investigated the effect of ACL remnant cell/BMSC co‐culture medium, along with its main activator, which simulates the ACL remnant tissue covered with BMSCs during ACL reconstruction. Notably, CM significantly enhanced the proliferation, migration, collagen synthesis and tenogenesis capabilities of both the ACL remnant cells and BMSCs. Furthermore, the contrasting effects of CM ∆ EVs treatment confirmed EVs as the major active ingredient in the CM underlying the beneficial effects of the co‐culture medium. The CM‐EV uptake by the ACL remnant cells and BMSCs significantly enhanced cell viability, proliferation, migration and expression of collagen synthesis‐, *TGF‐β*‐, *VEGF*‐ and tenogenesis‐related genes compared with BMSC‐EV and non‐EV uptakes.

In the intraarticular microenvironment, the ACL remnant tissue is coated with bone marrow from the drilled bone tunnel and the mixture surrounds the hamstring tendon graft during ACL reconstruction. Notably, the ACL remnants exhibit the paracrine potential of regulating the surrounding BMSCs toward increased collagen synthesis and tenogensis.^12^ Furthermore, Lu et al.^13^ demonstrated that the ACL remnant cell/BMSC co‐culture medium simultaneously attenuated the apoptosis and enhanced the activity of the hamstring tendon and tenocyte. In the present study, we demonstrated that the co‐culture medium‐treated ACL remnant cells and BMSCs presented increased cell activity and gene expression capability. In summary, the ACL remnant cell/BMSC co‐culture medium promotes the activity of hamstring tendon and tenocytes and also provides feedback to enhance ACL remnant cells and BMSCs activities. Therefore, the mixture of ACL remnant and bone marrow cells may improve remnant preservation and promote implanted tendon graft maturation during ACL reconstruction.

The co‐culture systems (indirect and direct) were introduced in the laboratory studies to observe the cell–cell interactions. A previous study, using the indirect co‐culture system, demonstrated the paracrine effects and BMSC upregulation (lower chamber) by ACL remnant cells (upper chamber).^12^ However, the indirect co‐culture system reflects only unidirectional signal transmission but not the bidirectional response to the ACL remnant cells and BMSCs in the intraarticular microenvironment during ACL reconstruction. In a direct co‐culture system, cell–cell interactions occur through direct gap junctions and autocrine, paracrine or endocrine signalling.^25^, ^26^ Moreover, during cell–cell interaction, various secretomes present in the medium transfer different signals.^25^, ^26^ Among these secretomes, EVs play an important role in cellular communication and signal transfer. In this study, we directly co‐cultured the ACL remnant cells and BMSCs to mimic the clinical environment of ACL reconstruction. The ACL remnant cell/BMSC co‐culture medium showed high capability to bidirectionally enhance the ACL remnant cells and BMSCs, which declined significantly after the removal of the EVs. These results indicated that CM‐EV facilitated the interactions between the ACL remnant cells and BMSCs in the direct co‐culture system. Therefore, EVs were identified as the main activators within the co‐culture medium.

EVs, containing variable mRNAs, microRNAs, lipids and proteins, derived from the stem cell culture medium exhibit a high potential to enhance injured tissue repair and cellular activity with a synergic effect.^14^, ^15^, ^27^, ^28^ Consequently, they can be used as a suitable alternative for treating cartilage injury, bone defect and tendon/ligament injury healing.^18^, ^28^, ^29^, ^30^, ^31^ Qi et al.^29^ investigated a novel purified exosome product and found that the tenocytes could uptake these exosomes with increasing cell proliferation, tenogenic marker expression and collagen deposition. Furthermore, Shi et al.,^30^ applying the purified exosome product patch in a tendon‐repair ex vivo model, demonstrated that it enhanced ultimate failed strength, reduced healing gap, increased *COL‐III* gene expression and decreased inflammatory response. In the rat patella tendon defect study, EV treatment enhanced tendon healing by reducing inflammation and tenocyte apoptosis, increasing tenogenic progenitor cell proportion, enhancing the expression of collagen and tenogenic marker genes, and improving histological collagen alignment.^31^ In our study, we harvested EVs from the BMSC‐only culture medium and ACL remnant cell/BMSC co‐culture medium. Treatment with these EVs increased the cell viability, proliferation, migration and gene expression of collagen synthesis, *TGF‐β*, *VEGF* and tenogenic markers in ACL remnant cells and BMSCs. Moreover, CM‐EV exhibited a higher potential to enhance the activities of ACL remnant cells and BMSCs compared with BMSC‐EV. Nevertheless, EV components are not static, and their production varies depending on stimulation conditions, culture environments and cell interactions.^25^, ^32^ In this study, we used the EV‐free medium in all culture conditions to eliminate the interference of the EV‐containing conventional medium. The EVs released following the crosstalk between ACL remnant cells and BMSCs exhibited higher potential for increasing cell activity within the microenvironment of ACL reconstruction compared with those released from the BMSC‐only medium. Therefore, CM‐EV may be used in ACL reconstruction to improve ACL remnant cells and BMSCs activities and consequently enhance graft maturation.

The present study has some limitations. First, we only investigated the effects of EVs on ACL remnant cells and BMSCs in vitro, and the enhancing capability of CM‐EV should be validated using animal studies. Second, the components within the ACL remnant cell/BMSC co‐culture medium were not evaluated. This study investigates EVs extracted from ACL cells/BMSCs co‐culture and BMSCs culture, focusing on their impact on cellular activities.^33^, ^34^, ^35^ EVs are composed of various components such as proteins, microRNAs and lipids, each extensively studied in the literature. However, there is no definitive conclusion regarding whether the effects of EVs arise from individual components or from their synergistic interactions.^36^, ^37^ Identifying specific EV components and their mechanisms of action would necessitate a separate, more comprehensive research effort, which represents a limitation of the current study. Third, we performed direct monolayer co‐culture using ACL remnant cells and BMSCs. Consequently, further studies using a 3D co‐culture system are essential to clarifying the relationship between ACL remnant cells, bone marrow cells and tendon grafts.

In conclusion, under the coexistence of ACL remnant cells and BMSCs, the secreted EVs increase cell viability, proliferation, migration and gene expression of *COL‐I* & *III*, *TGF‐β* and *TNC* in both cell types, which may be beneficial for the maturation of the implanted graft in ACL reconstruction. These findings hold high translational potential, offering insights and applications that could directly benefit patients with ACL reconstruction. The EVs derived from the co‐culture medium could be a potential biomaterial to improve graft maturation following ACL reconstruction.

## AUTHOR CONTRIBUTIONS

**Sung‐Yen Lin:** Conceptualization (equal); data curation (equal); formal analysis (supporting); funding acquisition (supporting); investigation (equal); methodology (supporting); project administration (supporting); writing – original draft (equal). **Shun Cheng Wu:** Conceptualization (supporting); data curation (supporting); formal analysis (equal); investigation (equal); methodology (supporting); writing – original draft (supporting). **Zi‐Miao Liu:** Conceptualization (supporting); data curation (supporting); formal analysis (supporting); investigation (equal); methodology (equal); software (supporting); writing – original draft (supporting). **Paul Pei‐Hsi Chou:** Investigation (supporting); supervision (equal); validation (equal). **Chunfeng Zhao:** Conceptualization (supporting); investigation (supporting); methodology (supporting); supervision (supporting). **Mei‐Ling Ho:** Investigation (supporting); methodology (supporting); resources (supporting); supervision (equal); validation (equal). **Cheng‐Chang Lu:** Conceptualization (equal); data curation (equal); formal analysis (equal); funding acquisition (equal); investigation (equal); methodology (equal); project administration (equal); software (equal); supervision (equal); validation (equal); visualization (equal).

## CONFLICT OF INTEREST STATEMENT

Cheng‐Chang Lu and all authors, or any member of his or her immediate family has no funding or commercial associations (e.g. consultancies, stock ownership, equity interest, patent/licensing arrangements) that might pose a conflict of interest in connection with the submitted article. I agree and confirm that this statement is true.

## Supporting information


Table S1.

## Data Availability

The data that support the findings of this study are available from the corresponding author upon reasonable request.

## References

[1] Bradley JP , Klimkiewicz JJ , Rytel MJ , Powell JW . Anterior cruciate ligament injuries in the National Football League: epidemiology and current treatment trends among team physicians. Art Ther. 2002;18(5):502‐509. 10.1053/jars.2002.30649 11987061

[2] Gianotti SM , Marshall SW , Hume PA , Bunt L . Incidence of anterior cruciate ligament injury and other knee ligament injuries: a national population‐based study. Sci Med Sport. 2009;12(6):622‐627. 10.1016/j.jsams.2008.07.005 18835221

[3] Sanders TL , Maradit Kremers H , Bryan AJ , et al. Incidence of anterior cruciate ligament tears and reconstruction: a 21‐year population‐based study. Am J Sports Med. 2016;44(6):1502‐1507. 26920430 10.1177/0363546516629944

[4] Janssen RP , van der Wijk J , Fiedler A , Schmidt T , Sala HA , Scheffler SU . Remodelling of human hamstring autografts after anterior cruciate ligament reconstruction. Knee Surg Sports Traumatol Arthrosc. 2011;19(8):1299‐1306. 21293848 10.1007/s00167-011-1419-y PMC3136699

[5] Janssen RP , Scheffler SU . Intra‐articular remodelling of hamstring tendon grafts after anterior cruciate ligament reconstruction. Knee Surg Sports Traumatol Arthrosc. 2014;22(9):2102‐2108. 23982759 10.1007/s00167-013-2634-5 PMC4142140

[6] Hong L , Li X , Zhang H , et al. Anterior cruciate ligament reconstruction with remnant preservation: a prospective, randomized controlled study. Am J Sports Med. 2012;40(12):2747‐2755. 23075805 10.1177/0363546512461481

[7] Hu J , Qu J , Xu D , Zhang T , Zhou J , Lu H . Clinical outcomes of remnant preserving augmentation in anterior cruciate ligament reconstruction: a systematic review. Knee Surg Sports Traumatol Arthrosc. 2014;22(9):1976‐1985. 24185826 10.1007/s00167-013-2749-8

[8] Song GY , Zhang J , Li X , Li Y , Feng H . Biomechanical and biological findings between acute anterior cruciate ligament reconstruction with and without an augmented remnant repair: a comparative in vivo animal study. Art Ther. 2016;32(2):307‐319. 10.1016/j.arthro.2015.08.011 26474744

[9] Matsumoto T , Ingham SM , Mifune Y , et al. Isolation and characterization of human anterior cruciate ligament‐derived vascular stem cells. Stem Cells Dev. 2012;21(6):859‐872. 21732814 10.1089/scd.2010.0528 PMC3871494

[10] Matsumoto T , Kubo S , Sasaki K , et al. Acceleration of tendon‐bone healing of anterior cruciate ligament graft using autologous ruptured tissue. Am J Sports Med. 2012;40(6):1296‐1302. 22427618 10.1177/0363546512439026

[11] Mifune Y , Matsumoto T , Ota S , et al. Therapeutic potential of anterior cruciate ligament‐derived stem cells for anterior cruciate ligament reconstruction. Cell Transplant. 2012;21(8):1651‐1665. 22732227 10.3727/096368912X647234

[12] Lu CC , Chou SH , Shen PC , Chou PH , Ho ML , Tien YC . Extracorporeal shock wave promotes activation of anterior cruciate ligament remnant cells and their paracrine regulation of bone marrow stromal cells' proliferation, migration, collagen synthesis, and differentiation. Bone Joint Res. 2020;9(8):458‐468. 32832074 10.1302/2046-3758.98.BJR-2019-0365.R1 PMC7418778

[13] Lu CC , Ho CJ , Chen SJ , et al. Anterior cruciate ligament remnant preservation attenuates apoptosis and enhances the regeneration of hamstring tendon graft. Bone Joint Res. 2023;12(1):9‐21. 36617435 10.1302/2046-3758.121.BJR-2021-0434.R2 PMC9872040

[14] Camussi G , Deregibus MC , Cantaluppi V . Role of stem‐cell‐derived microvesicles in the paracrine action of stem cells. Biochem Soc Trans. 2013;41(1):283‐287. 23356298 10.1042/BST20120192

[15] Toh WS , Lai RC , Zhang B , Lim SK . MSC exosome works through a protein‐based mechanism of action. Biochem Soc Trans. 2018;46(4):843‐853. 29986939 10.1042/BST20180079 PMC6103455

[16] Keshtkar S , Azarpira N , Ghahremani MH . Mesenchymal stem cell‐derived extracellular vesicles: novel frontiers in regenerative medicine. Stem Cell Res Ther. 2018;9(1):63. 29523213 10.1186/s13287-018-0791-7 PMC5845209

[17] Zhang L , Jiao G , Ren S , et al. Exosomes from bone marrow mesenchymal stem cells enhance fracture healing through the promotion of osteogenesis and angiogenesis in a rat model of nonunion. Stem Cell Res Ther. 2020;11(1):38. 31992369 10.1186/s13287-020-1562-9 PMC6986095

[18] Liu X , Yang Y , Li Y , et al. Integration of stem cell‐derived exosomes with in situ hydrogel glue as a promising tissue patch for articular cartilage regeneration. Nanoscale. 2017;9(13):4430‐4438. 28300264 10.1039/c7nr00352h

[19] Liu WC , Chen CT , Lu CC , et al. Extracorporeal shock wave therapy shows superiority over injections for pain relief and grip strength recovery in lateral epicondylitis: a systematic review and network meta‐analysis. Art Ther. 2022;38(6):2018‐2034.e12. 10.1016/j.arthro.2022.01.025 35093494

[20] Rosenthal AK , Gohr CM , Mitton‐Fitzgerald E , et al. Autophagy modulates articular cartilage vesicle formation in primary articular chondrocytes. J Biol Chem. 2015;290(21):13028‐13038. 25869133 10.1074/jbc.M114.630558 PMC4505557

[21] Eirin A , Riester SM , Zhu XY , et al. MicroRNA and mRNA cargo of extracellular vesicles from porcine adipose tissue‐derived mesenchymal stem cells. Gene. 2014;551(1):55‐64. 25158130 10.1016/j.gene.2014.08.041 PMC4174680

[22] Vis MAM , Ito K , Hofmann S . Impact of culture medium on cellular interactions in in vitro co‐culture systems. Front Bioeng Biotechnol. 2020;8:911. 32850750 10.3389/fbioe.2020.00911 PMC7417654

[23] Kook YM , Jeong Y , Lee K , Koh WG . Design of biomimetic cellular scaffolds for co‐culture system and their application. J Tissue Eng. 2017;8:2041731417724640. 29081966 10.1177/2041731417724640 PMC5564857

[24] Lai RC , Tan SS , Teh BJ , et al. Proteolytic potential of the MSC exosome proteome: implications for an exosome‐mediated delivery of therapeutic proteasome. Int J Proteomics. 2012;2012:971907. 22852084 10.1155/2012/971907 PMC3407643

[25] Toh WS , Lai RC , Hui JHP , Lim SK . MSC exosome as a cell‐free MSC therapy for cartilage regeneration: implications for osteoarthritis treatment. Semin Cell Dev Biol. 2017;67:56‐64. 27871993 10.1016/j.semcdb.2016.11.008

[26] Phinney DG , Pittenger MF . Concise review: MSC‐derived exosomes for cell‐free therapy. Stem Cells. 2017;35(4):851‐858. 28294454 10.1002/stem.2575

[27] Zhang S , Chu WC , Lai RC , Lim SK , Hui JH , Toh WS . Exosomes derived from human embryonic mesenchymal stem cells promote osteochondral regeneration. Osteoarthr Cartil. 2016;24(12):2135‐2140. 10.1016/j.joca.2016.06.022 27390028

[28] Furuta T , Miyaki S , Ishitobi H , et al. Mesenchymal stem cell‐derived exosomes promote fracture healing in a mouse model. Stem Cells Transl Med. 2016;5(12):1620‐1630. 27460850 10.5966/sctm.2015-0285 PMC5189643

[29] Qi J , Liu Q , Reisdorf RL , et al. Characterization of a purified exosome product and its effects on canine flexor tenocyte biology. J Orthop Res. 2020;38(8):1845‐1855. 31930553 10.1002/jor.24587

[30] Shi G , Wang Y , Wang Z , et al. A novel engineered purified exosome product patch for tendon healing: an explant in an ex vivo model. J Orthop Res. 2021;39(8):1825‐1837. 32936480 10.1002/jor.24859 PMC9235100

[31] Shi Z , Wang Q , Jiang D . Extracellular vesicles from bone marrow‐derived multipotent mesenchymal stromal cells regulate inflammation and enhance tendon healing. J Transl Med. 2019;17(1):211. 31238964 10.1186/s12967-019-1960-x PMC6593555

[32] Ferreira JR , Teixeira GQ , Santos SG , Barbosa MA , Almeida‐Porada G , Gonçalves RM . Mesenchymal stromal cell secretome: influencing therapeutic potential by cellular pre‐conditioning. Front Immunol. 2018;9:2837. 30564236 10.3389/fimmu.2018.02837 PMC6288292

[33] Qiu G , Zheng G , Ge M , et al. Functional proteins of mesenchymal stem cell‐derived extracellular vesicles. Stem Cell Res Ther. 2019;10(1):359. 31779700 10.1186/s13287-019-1484-6 PMC6883709

[34] Foo JB , Looi QH , How CW , et al. Mesenchymal stem cell‐derived exosomes and MicroRNAs in cartilage regeneration: biogenesis, efficacy, miRNA enrichment and delivery. Pharmaceuticals (Basel). 2021;14(11):1093. 34832875 10.3390/ph14111093 PMC8618513

[35] Record M , Silvente‐Poirot S , Poirot M , Wakelam MJO . Extracellular vesicles: lipids as key components of their biogenesis and functions. J Lipid Res. 2018;59(8):1316‐1324. 29764923 10.1194/jlr.E086173 PMC6071772

[36] Liu YJ , Wang C . A review of the regulatory mechanisms of extracellular vesicles‐mediated intercellular communication. Cell Commun Signal. 2023;21(1):77. 37055761 10.1186/s12964-023-01103-6 PMC10100201

[37] Tsai YC , Cheng TS , Liao HJ , et al. Mesenchymal stem cell secreted‐extracellular vesicles are involved in chondrocyte production and reduce adipogenesis during stem cell differentiation. Tissue Eng Regen Med. 2022;19(6):1295‐1310. 36346531 10.1007/s13770-022-00490-0 PMC9679102

