# Supplementary material for: Bone marrow stromal and anterior cruciate ligament remnant cell co‐culture‐derived extracellular vesicles promote cell activity in both cell types
Source: J Cell Mol Med. 2024 Sep 1;28(17):e70049. doi: 10.1111/jcmm.70049 (PMC11366498; doi:10.1111/jcmm.70049)
Supplement: Supplementary file 1 — Table S1. [file JCMM-28-e70049-s001.docx]

Supplementary Table 1. Primers sequences used for complementary DNA synthesis

| **Gene name** | **Amplicon size, bp** | **Primer sequence** | |
| --- | --- | --- | --- |
| *COL-1* | 73 | Forward | 5ʹ-TTCTGCAGGGCTCCAATGA-3ʹ |
|  |  | Reverse | 5ʹ-TCGACAAGAACAGTGTAAGTGAACCT-3ʹ |
| *COL-III* | 92 | Forward | 5ʹ-CCTGAAGCCCCAGCAGAA-3ʹ |
|  |  | Reverse | 5ʹ-AACAGAAATTTAGTTGGTCACTTGTACTG-3ʹ |
| *TGF-β* | 140 | Forward | 5ʹ-CAGTGGAAAGACCCCACATCTC-3ʹ |
|  |  | Reverse | 5ʹ-GACGCAGGCAGCAATTATCC-3ʹ |
| *VEGF* | 122 | Forward | 5ʹ-ATCATGCGGATCAAACCTCA-3ʹ |
|  |  | Reverse | 5ʹ-CAAGGCCCACAGGGATTTTC-3ʹ |
| *Scx* | 178 | Forward | 5ʹ-CCCAAACAGATCTGCACCTT-3ʹ |
|  |  | Reverse | 5ʹ-TCCATCTGTCCATCTGTCCA-3ʹ |
| *TNC* | 78 | Forward | 5ʹ-CAGAAGCCTTGGCCATGTG-3ʹ |
|  |  | Reverse | 5ʹ-GCACTCTCTCCCCTGTGTAGGA-3ʹ |
| *Ki67* | 192 | Forward | 5ʹ-ACACCGCTCAAAAGAGGAGA-3ʹ |
|  |  | Reverse | 5ʹ-ATCATTCGCAACTGGAGGAC-3ʹ |
